# Fatigue Life Prediction Model of FRP–Concrete Interface Based on Gene Expression Programming

**DOI:** 10.3390/ma17030690

**Published:** 2024-01-31

**Authors:** Zhimei Zhang, Yinglong Huo

**Affiliations:** Department of Civil Engineering, School of Mechanics and Engineering Science, Shanghai University, Shanghai 200444, China; zhangzhimei@staff.shu.edu.cn

**Keywords:** gene expression programming, FRP–concrete interface, fatigue life, prediction model

## Abstract

Under fatigue loading, the interfacial fatigue life of fiber-reinforced polymer(FRP)–concrete is an important index for the analysis of the fatigue performance of reinforced concrete beams strengthened with FRP materials and the evaluation of the reinforcement effect. To solve the problems of the inconsistent and limited accuracy of existing fatigue life prediction models, gene expression programming (GEP) was used to study the interfacial fatigue life of FRP–concrete. Firstly, 219 sets of interfacial fatigue test data were collected, which included two kinds of reinforcement methods, namely, externally bonded (EB) reinforcement and near-surface-mounted (NSM) reinforcement; secondly, Pearson correlation analysis was used to determine the key factors affecting the fatigue life, and then GEP was used to explore the influence of different input forms on the prediction accuracy of the model. Fatigue life calculation formulas applicable to the two kinds of reinforcement methods, i.e., EB and NSM, were established, and a specific calculation formula was established. The model was subjected to parameter sensitivity analysis and variable importance analysis and was found to reflect the intrinsic relationship between the fatigue life and various factors. Finally, the GEP model was compared with the models proposed by other researchers. Five statistical indices, such as the coefficient of determination and the average absolute error, were selected to assess the model, and the results show that the GEP model has higher prediction accuracy than other models, with a coefficient of determination of 0.819, and indicators such as the average absolute error are also lower than those of the rest of the models.

## 1. Introduction

In recent years, fiber-reinforced polymer (FRP) has played an important role in the structural reinforcement of bridges due to its advantages of being light in weight, high in strength, fatigue-resistant, and convenient for construction [1,2,3], such as in the context of externally bonded (EB) FRP reinforcement and near-surface-mounted (NSM) FRP reinforcement [4]. However, under vehicle fatigue loading, the degradation of FRP–concrete interfacial bonding performance of the reinforced bridges will be gradually aggravated, resulting in a continuous decrease in the bearing capacity and fatigue life of the reinforced bridges. The interfacial bonding performance of FRP–concrete is an important factor affecting the reinforcing effect of the reinforced bridges, and the fatigue life of the FRP–concrete interface is an important index for evaluating the bonding performance of the interface. However, due to the difficulty of observing the interface test phenomenon and the long test time, research on the fatigue performance of FRP–concrete interfacial bonds is still limited. Therefore, it is necessary to study the interfacial fatigue life and provide a reference for the fatigue design of FRP-reinforced concrete beams.

Many scholars have conducted extensive experimental studies on the fatigue performance of the FRP–concrete interface and explored the variation rule of the interface fatigue life with the main test variables. Ma et al. [5] found that increasing the CFRP’s bond length and the number of bond layers, increasing the concrete strength, or decreasing the loading frequency can improve the fatigue life of the specimens through single-shear pull-out tests. Bizindavyi et al. [6] found that increasing the FRP bond length and width can improve the interfacial fatigue life through single-shear pull-out tests and established interfacial fatigue life curves (S–N curves) for CFRP and GFRP, respectively, with different bond lengths. Li et al. [7] used modified beam tests to investigate the interfacial fatigue properties of CFRP–concrete that were more in line with the actual stress state. They pointed out that the interfacial fatigue life increases with the increase in concrete strength and CFRP bond length, and decreases with the rise in the CFRP-to-concrete width ratio and the loading frequency. They also proposed empirical formulas for predicting the interfacial fatigue life considering four factors: the stress level, the compressive strength of the concrete, the CFRP-to-concrete width ratio, and the bond length. Zhu et al. [8] found that the interfacial fatigue life increases with increasing concrete strength based on modified beam tests, and they proposed a model for fatigue life prediction considering the stress level and the concrete strength. Xie et al. [9] presented an empirical equation for the fatigue life of the interface of the reinforced specimen and the upper limit of the FRP fatigue stress in the span through a four-point bending loading test, and they found that the fatigue life of the specimen reinforced with CFRP was significantly higher than that of the specimen reinforced with BFRP. Chalot et al. [10] obtained a model for fatigue life prediction based on the double-shear pull-out test and compared it with the model for interfacial fatigue life prediction proposed in the literature [7]. Min [11] investigated the effect of the fatigue load amplitude (the difference between the upper and lower limits of fatigue load) and the fatigue load level (the mean value of the upper and lower limits of fatigue load) on fatigue life using the single-shear pull-out test, which showed that the fatigue life decreases with the increase in the fatigue load amplitude and the fatigue load level. Fathi et al. [12] investigated the effects of composite type, CFRP-to-concrete width ratio, and bond length on the interfacial fatigue adhesion performance using double-shear pull-out tests. The results showed that the fatigue life of fabric sheet CFRP-reinforced concrete was larger than that of laminated CFRP-reinforced concrete and that the interfacial fatigue life increased with the increase in the bond length and the CFRP-to-concrete width ratio. In addition, they proposed an improved model for fatigue life prediction. Al-Saadi et al. [13] investigated the effect of CFRP strip dimensions and surface roughness on the interfacial bonding performance using a single-shear pull-out test and found that the fatigue life of CFRP specimens with a larger cross-section size and a rougher surface was higher. In addition, they modeled the S-N curves for different combinations of CFRP strip dimensions and surface roughness. Chou et al. [14] obtained an S–N curve in the case of interfacial stripping damage based on a single-shear pull-out test and proposed a model that can predict the relationship between fatigue stress amplitude, bond length, and fatigue life.

In summary, due to the difficulty of the FRP–concrete interface fatigue test, discrete test data, relatively single test variables, etc., the fatigue life of the interface with the main test variables of the change rule has not yet reached a consistent conclusion. Existing models for fatigue life prediction suffer from unstable prediction accuracy, limited generalization ability, a lack of a unified calculation model for different reinforcement methods, etc. Machine learning can process a large number of data and extract useful information and laws from them, thus providing accurate prediction models to overcome the limitations that traditional models face, and it has also shown better adaptability and generalization ability. Therefore, in this paper, 219 sets of FRP–concrete interfacial fatigue test data were collected to study the interfacial fatigue life. The collected data were analyzed using gene expression programming (GEP) to obtain the explicit expression between interfacial fatigue life and its influencing factors and to compare it with the models proposed by other researchers to verify its accuracy and reliability.

## 2. Gene Expression Programming

Gene expression programming is an evolutionary algorithm based on the idea of biological heredity, which firstly constructs a certain number of initial populations, secondly evaluates the fitness of individuals according to the fitness function, then selects the optimal individuals and forms new populations through genetic mutation using genetic operators, and finally iterates this process until the termination conditions are reached. This algorithm combines the fixed-length linear chromosomes of the Genetic Algorithm (GA) with the expression tree of Genetic Programming (GP) with different shapes and sizes. This method can obtain the functional relationship between the predicted variables and the input parameters. Most machine learning models simply take inputs and give outputs and are often regarded as “black box” models whose decision-making process involves a large number of hidden layers and nonlinear transformations, which makes it difficult to directly explain their inner workings (although Zhang et al. [15] provide a method to transform ANN models into mathematical formulas in a way that reduces the prediction accuracy of the original ANN models). In contrast, gene expression programming can directly obtain computational formulas that can be used for engineering applications, thus solving problems that cannot be explained by other machine learning models while maintaining accuracy.

Chromosomes are composed of genes that control different traits, and genes in gene expression programming consist of a head and a tail, where the head determines the function and behavior of the gene, while the tail structure is mainly used to control the replication and mutation of the gene. By combining, mutating, and selecting the head and tail, gene expression programming can solve and optimize the problem. To solve problems, the head length can be determined first, and then the tail length can be determined using Equation (1).

(1) $$t=h\times \left(n-1\right)+1$$

where t is the length of the gene tail, h is the length of the gene head, and n is the maximum number of operands in the function set.

**Figure 1:**
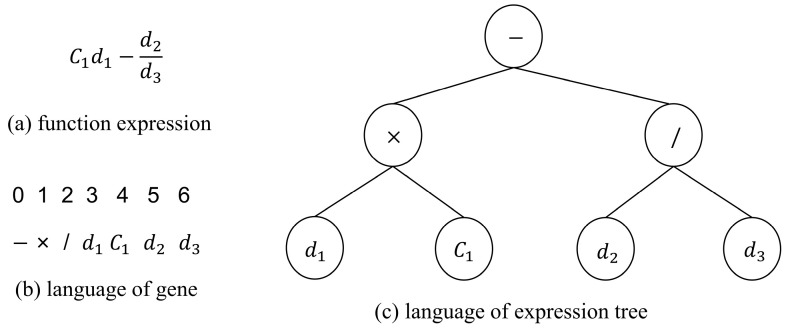
Diagram of the GEP language.

The two languages in gene expression programming are the language of the expression tree and the language of the gene. The language of the expression tree is a language that uses a tree structure to represent a function, in which the inner nodes of the tree represent the set of function symbols and the leaf nodes represent the set of endpoint symbols. In the language of the gene, each gene element corresponds to an operation (e.g., addition, subtraction, or logical operation) or parameter (e.g., a constant or a value of a variable) in a function. Figure 1 shows the mutual conversion method of the two languages. By combining genes in a specific way, the corresponding expression tree can be obtained. Furthermore, by traversing the expression tree from top to bottom and from left to right, these can be converted into the corresponding gene elements. As shown in Figure 1, for example, it starts with the root node of the expression tree (subtraction operation) and then processes its left and right child nodes separately; the multiplication node on the left combines $C_{1}$ and $d_{1}$ to form sub-expression $C_{1}d_{1}$, while the division node on the right combines $d_{2}$ and $d_{3}$ to form sub-expression $d_{2}/d_{3}$ and finally combines the two sub-expressions by subtracting them through the root node to obtain the complete symbolic expression $C_{1}d_{1}-d_{2}/d_{3}$.

## 3. GEP-Based Fatigue Life Prediction Model for FRP–Concrete Interface

### 3.1. Experimental Data and Correlation Analysis

**Figure 2:**
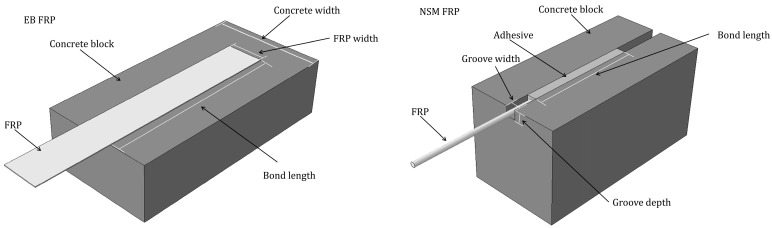
Schematic diagram of direct shear test.

A total of 219 sets of FRP–concrete interfacial fatigue shear test data were collected from the literature [10,11,12,13,14,16,17,18,19,20,21,22,23,24,25,26,27,28,29], including 108 sets of EB FRP–concrete interfacial fatigue tests and 111 sets of NSM FRP–concrete interfacial fatigue tests, and the direct shear tests of the two types of reinforcement are shown in Figure 2. It is worth noting that since bridge structures are generally subjected to traffic loads with a low load amplitude and generally fail under high weekly fatigue loads, the number of loading times before damage of the specimens selected in this paper are all greater than 1000 [30]. The FRP types include CFRP, GFRP, and AFRP, and other types of specimens are excluded. The test data are all from single-shear tests, double-shear tests, or modified beam tests and did not consider the specimens with external anchorage systems. All the data were statistically analyzed, and the results are shown in Table 1, the full datebase refer to the Supplementary Materials.

In the table, $f_{t}$ is the tensile strength of concrete; L is the bond length of FRP; $A_{f}$ is the cross-sectional area of FRP; $E_{f}$ is the modulus of elasticity of FRP; w denotes the width of FRP in the EB FRP systems and the depth of the groove in the NSM FRP systems; W denotes the width of concrete in the EB FRP systems and the width of the groove in the NSM FRP systems; N is the number of cycles in the case of damage; $S_{max}$=$P_{max}/P_{u}$, and $S_{min}=P_{min}/P_{u},{\;S}_{a}$ = ($S_{max}$+$S_{min}$)/2, ∆S = $S_{max}{-S}_{min},{\;S=S}_{a}\times ∆S;\;P_{max},\;\;P_{min}$, $P_{u}$ are the upper fatigue load limit, lower fatigue load limit, and interfacial bond strength under static load conditions, respectively. It should be noted that, for the convenience of statistics and the derivation of equations, the compressive strength of concrete cubes and cylinders in the test database is unified with the expression $f_{t}$ in this paper, where the cylindrical compressive strength $f_{c}^{\prime }$ can be converted to cube compressive strength $f_{c}$ based on $f_{c}^{\prime }=0.8f_{c}$ [31] and then to tensile strength $f_{t}$ based on $f_{t}=0.26\sqrt[{3}]{{f_{c}}^{2}}$ [32].

In order to solve the correlation between the variables in Table 1 and between the variables and fatigue life, it is necessary to carry out a Pearson correlation analysis on the data in Table 1. The Pearson correlation coefficient, denoted as r, assesses the linear correlation between two continuous variables and is calculated using the formula:

(2) $$r=\frac{\sum_{i}^{n}\left(x_{i}-\bar{x})\times (y_{i}-\bar{y}\right)}{\sqrt{\sum_{i}^{n}{\left(x_{i}-\bar{x}\right)}^{2}}\times \sqrt{\sum_{i}^{n}{\left(y_{i}-\bar{y}\right)}^{2}}}$$

where r is the Pearson correlation coefficient, $x_{i},\;y_{i}$ are the experimental values of the two variables, and $\bar{x},\;\bar{y}$ are the mean values of the two variables.

**Figure 3:**
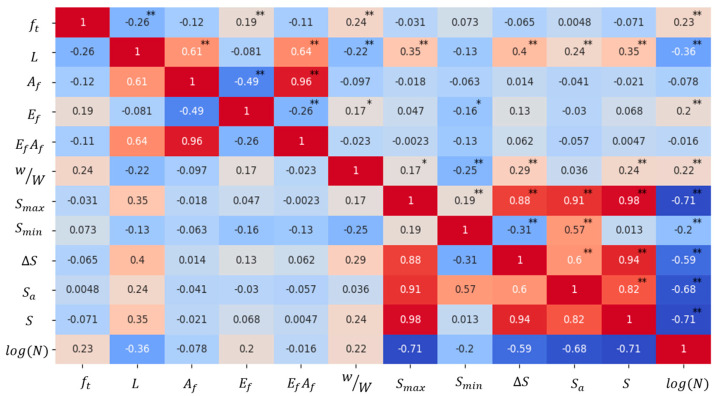
Pearson correlation coefficient.

The results of Pearson correlation analysis are shown in Figure 3, where the color depth of each cell reflects the magnitude of the correlation of the variables: dark red indicates a strong positive correlation, dark blue indicates a strong negative correlation, and the color tends to be white to indicate a weak correlation. Correlation coefficients range from +1 (perfect positive correlation) to −1 (perfect negative correlation), with 0 indicating no linear relationship. In the graph, ** indicates that two variables are significantly correlated at the 0.01 level, meaning there is a 99% confidence level of a significant linear relationship, and * signifies that two variables are significantly correlated at the 0.05 level, indicating a 95% confidence level that there is a statistically significant linear relationship between the two variables.

From the figure, it can be seen that the fatigue life is significantly correlated at the 0.01 level with parameters such as $f_{t},\;L,{\;E}_{f},\;w/W,$ and S, so we can focus on considering these significantly correlated parameters as input parameters for the model. In addition, there is the problem of multicollinearity in the regression problem based on machine learning [15]. If the correlation coefficient of two variables is greater than 0.80, there is considered to be a strong correlation, and when there is a strong correlation between the independent variables, it will affect the explanatory and predictive ability of the model. As can be seen from Figure 3, the correlation between $f_{t},\;L,\;E_{f},\;w/W$, and S is low, so there is no risk of a multicollinearity problem.

### 3.2. GEP Model Parameter Settings

The GeneXproTools 5.0 software developed by Gepsoft was used to establish the model for the fatigue life prediction of the FRP–concrete interface, and the main steps are shown below.

**Figure 4:**
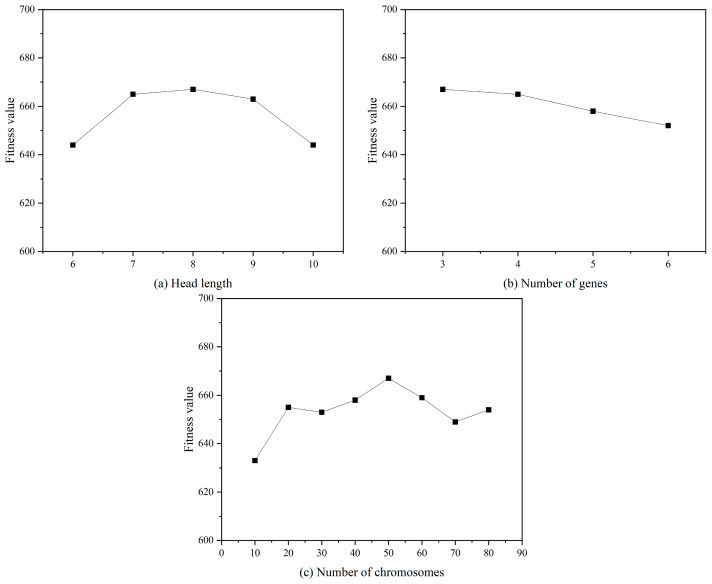
Determination of optimal parameters of the GEP model.

1. Selection of the test set and training set: In this paper, 219 sets of data were randomly divided into the training set and the test set at a ratio of 3:1, and 165 sets of training set data and 54 sets of test set data were obtained.
2. Selection of the fitness function: In this paper, we adopted the root-mean-squared error (RMSE) as the optimization objective, which quantifies the gap between the prediction result of an individual and the actual target, and we selected the population by calculating the RMSE as follows:
  (3) $$f_{i}=\frac{1000}{1+\sqrt{\frac{1}{n}\sum_{i=1}^{n}{\left(y_{i}-y_{i,j}\right)}^{2}}}$$
  In the equation, $f_{i}$ denotes the fitness value, n denotes the total number of chromosomes, $y_{i}$ denotes the predicted value, and $y_{i,j}$ denotes the true value.

3. Determination of the endpoint set T and the function set F: The endpoint set T consists of numerical constants, variables to be solved, and uninvolved functions, and the function symbol set F consists of function symbols of the model expression. In this paper, F={+,–,×,/,X2,X3,Inv,3Rt}, in which X2,X3,Inv, and 3Rt represent, respectively, $x^{2},x^{3},1/x$, and $\sqrt[{3}]{x}$, and mathematical expressions can be constructed based on these function symbols.
4. Selection of the linkage function: The linkage function determines how to combine the genomes to form an effective gene expression. Commonly used linkage functions are addition (+), subtraction (−), multiplication (×), and division (/) [33]. In this study, better results can be obtained by choosing addition (+) as a linking function compared to other linking functions (−, ×, /), and the literature [34,35] yielded similar results.
5. Parameter setting: A trial-and-error strategy is used to determine the optimal values of gene head length (gene tail length is determined according to Equation (1)), gene number, and chromosome number. The change in fitness value with the gene head length, gene number, and chromosome number is shown schematically in Figure 4, and the value corresponding to the maximum fitness value is determined as the optimal value of this parameter. Obviously, the optimal values of gene head length, gene number, and chromosome number are 8, 3, and 50, respectively. The set of genetic operators is used to carry out the gene crossover, gene mutation, and other operations in the process of optimization of the gene expression programming algorithm, and the values of the genetic operator set in this paper are set according to the “optimal evolution” strategy in GeneXproTools 5.0 software. The values are shown in Table 2.

### 3.3. Selection of the Optimal Input Form

Since the original data of the test can be combined to form new data (FRP axial rigidity), the existing models for fatigue life prediction differ in the selection of stress levels. Hence, this paper focuses on considering the following five input forms according to the results of the Pearson correlation analysis and the research results of the existing models to search for the optimal input form of the prediction model, which is shown in Table 3 for the form of the parameter input. The input parameters of model A are all original data, the input parameters of model B consider the FRP axial stiffness with certain physical meaning as input parameters, and models C~E are selected to compare the stress levels of different forms, in which the input parameters of model E are selected according to the results of Pearson correlation analysis.

**Figure 5:**
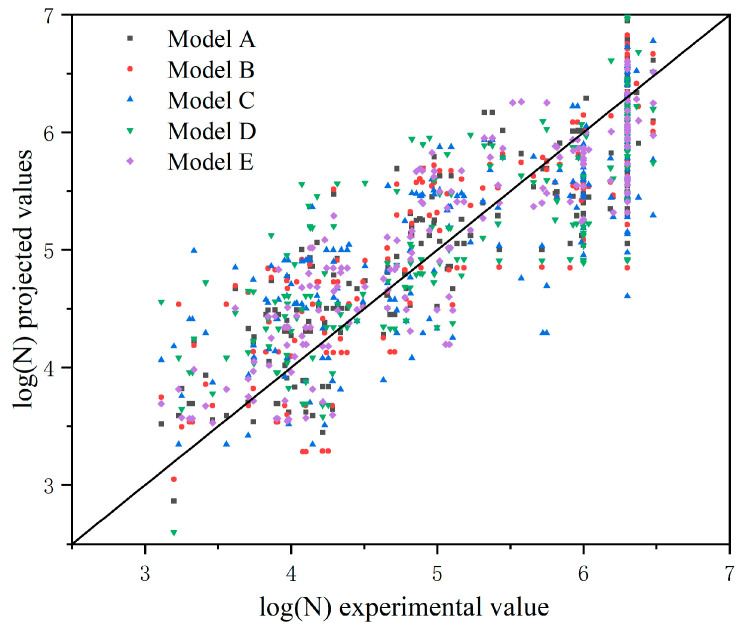
Comparison of the predicted and experimental values of interface fatigue life under different input forms.

By running GeneXproTools 5.0 according to the above parameter settings, the prediction models can be obtained under different input forms, and a comparison of the predicted and experimental values of the models for fatigue life prediction corresponding to the various input forms is shown in Figure 5. The models with different input forms were evaluated using the coefficient of determination ($R^{2}$), the mean absolute error (MAE), the root-mean-squared error (RMSE), the mean absolute percentage error (MAPE), and the relative square root error (RRSE), which were calculated using Equations (4) to (8), in which the higher the value of $R^{2}$, the smaller the values of RMSE, MAE, MAPE, and RRSE, indicating that the model’s prediction effect is better. The results of the calculation are shown in Table 4.

(4) $$R^{2}=\frac{{\left[\sum_{i=1}^{n}\left(x_{i}-\bar{x}\right)\left(y_{i}-\bar{y}\right)\right]}^{2}}{\sum_{i=1}^{n}{\left(x_{i}-\bar{x}\right)}^{2}\sum_{i=1}^{n}{\left(y_{i}-\bar{y}\right)}^{2}}$$

(5) $$\mathrm{RMSE}=\sqrt{\frac{1}{n}\sum_{i=1}^{n}{\left(x_{i}-y_{i}\right)}^{2}}$$

(6) $$\mathrm{MAE}=\frac{1}{n}\sum_{i=1}^{n}\left|x_{i}-y_{i}\right|$$

(7) $$\mathrm{RRSE}=\sqrt{\frac{\sum_{i=1}^{n}{\left(x_{i}-y_{i}\right)}^{2}}{\sum_{i=1}^{n}{\left(x_{i}-\bar{x}\right)}^{2}}}$$

(8) $$MAPE=\frac{1}{n}\sum_{i=1}^{n}\left|\frac{x_{i}-y_{i}}{x_{i}}\right|$$

where, $x_{i}$ is the experimental value, $y_{i}$ is the predicted value, $\bar{x}$ is the average of the experimental values, $\bar{y}$ is the average of the predicted values, and n is the total number of data.

**Figure 6:**
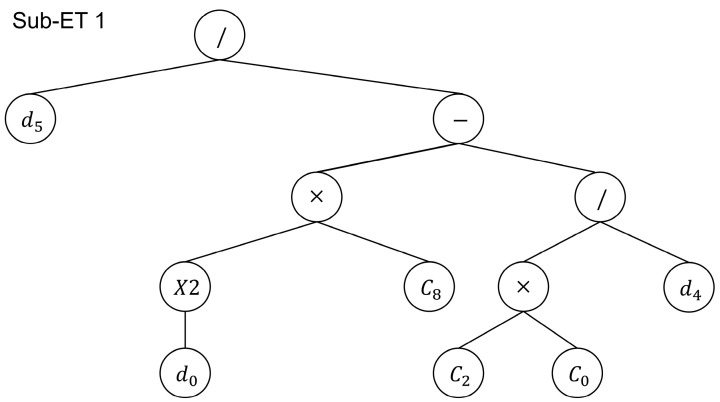

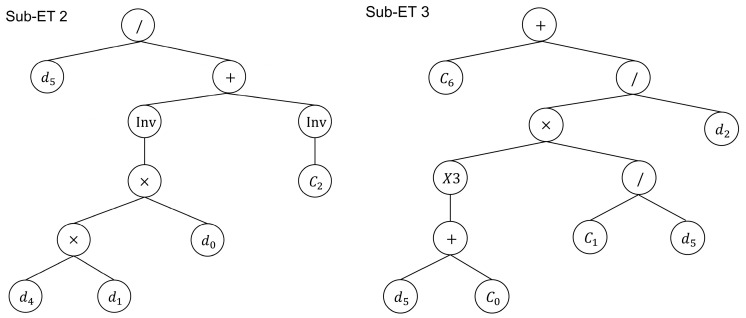
Expression tree.

As can be seen from Figure 5 and Table 4, the $R^{2}$ of Model E is 0.819, which is higher than that of the remaining four models, and its statistical indices, such as root-mean-squared error, are at the lowest level, indicating that the input form of Model E is the optimal input form among the above five models. Among them, Model A considers $A_{f}$ in the selection of independent variables rather than Model E, but its statistical indices are lower than those of Model E. The reason is that it is easy to produce overfitting with the increase in variable dimensions, which leads to the deterioration of its prediction effect. Model B uses FRP axial stiffness as an input parameter, and its prediction accuracy is not as good as that of Model E. This is because the correlation between FRP’s axial stiffness and fatigue life is much lower than the correlation between the FRP modulus of elasticity and fatigue life, which indicates that when choosing the input parameters of the model, the features with a higher correlation should be considered as input forms. Model E is renamed as the GEP model below, and its expression tree is shown in Figure 6, where $d_{0},d_{1},d_{2},d_{3},d_{4}$ represent the concrete tensile strength $f_{t}$, bond length L, FRP modulus of elasticity $E_{f}$, FRP-to-concrete width ratio (EB) or groove depth-to-width ratio (NSM) w/W, and loaded stress level S, respectively. 

Where C is a random constant, the C values in the first expression tree of this model $C_{0},C_{2},C_{8}$ are equal to 2.392, −4.279, and −0.119, respectively; the ones in the second expression tree $C_{2}$ are equal to −13.309; and the ones in the third expression tree $C_{0},C_{1},C_{6}$ are equal to 1.113, −6.6, and 8.31, respectively. The connecting function adopts the additive method (+) so that the fatigue life at the FRP–concrete interface of the prediction equation can be written as follows:

(9) $$\log\left(N\right)=8.31-\frac{{6.6(S+1.113)}^{3}}{SE_{f}}+\frac{S}{\frac{10.23}{w/W}-0.119{f_{t}}^{2}}+\frac{S}{\frac{1}{w/Wf_{t}L}-0.075}$$

### 3.4. Performance Evaluation of the Model

#### 3.4.1. Sensitivity Analysis of the Model

**Figure 7:**
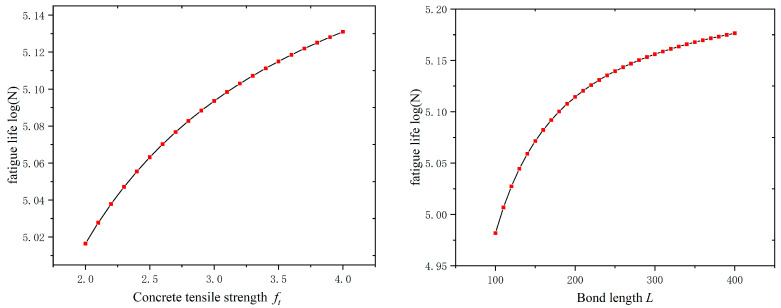

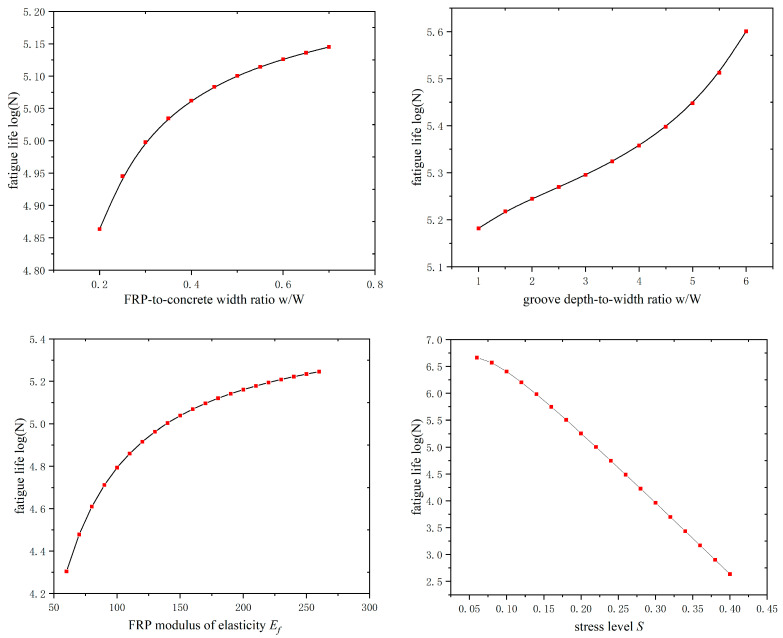
Sensitivity analysis results of GEP model for interface fatigue life.

To test whether the GEP model can correctly reflect the relationship between the factors and the fatigue life of the interface, parametric sensitivity analyses should be performed on the model. When investigating the effect of a factor on fatigue life, the median of the rest of the factors was taken, and the input variables were taken as follows: $f_{t}$ = 3.14 MPa, L = 180 mm, $E_{f}$ = 212 MPa, w/W = 1, and S = 0.21. The analysis results are shown in Figure 7. 

As can be seen in Figure 7, the fatigue life increases with the increase in the concrete tensile strength, the FRP modulus of elasticity, and the bond length, and it decreases with the increase in stress level, which is in agreement with the literature [5,7,8,9,18]. Min et al. [11] concluded that the fatigue life decreases with the increase in fatigue load amplitude, where the expression form of the stress level considers the effects of both relative fatigue load amplitude and relative fatigue load level. In addition, when the interface is subjected to the same fatigue load amplitude, the fatigue life decreases with the increase in the relative fatigue load level, which is consistent with the results of this paper. With the increase in the stress level, the fatigue damage mode will be transformed from the FRP debonding from the epoxy to the damage of the concrete cover separation [8], so it can be seen that improving the strength of concrete will also improve the interfacial fatigue life. The debonding between FRP and concrete is carried out progressively along the FRP bond layer with the number of loading times so that increasing the FRP bond’s length will also improve the interface’s fatigue life. For EB FRP, since there are fewer experimental studies on the fatigue life of FRP-to-concrete width ratio, no consistent conclusion has been drawn. Both this paper and the literature [6,12] studying the effect of FRP-to-concrete width ratio on the fatigue life have concluded that the interfacial fatigue life increases with the increase in the ratio of the width of FRP and concrete. It is worth stating that when studying the relationship between the groove’s depth-to-width ratio and the fatigue life, it only makes sense to control the groove depth or width to be constant. Al-Saadi et al.’s [13,28] study on the effect of the FRP cross-section size on fatigue life showed that, in conditions where the groove width is unchanged, the use of a larger cross-section size of the FRP often needs a larger depth of groove to increase the adhesion with the concrete, so the fatigue life increases with the increase in the groove depth-to-width ratio, and more tests need to be carried out to verify its effect on fatigue life. From the above analysis, it can be seen that the prediction model based on GEP can reflect the fatigue life and the intrinsic mechanism of each influencing factor.

#### 3.4.2. Analysis of the Importance of Variables

**Figure 8:**
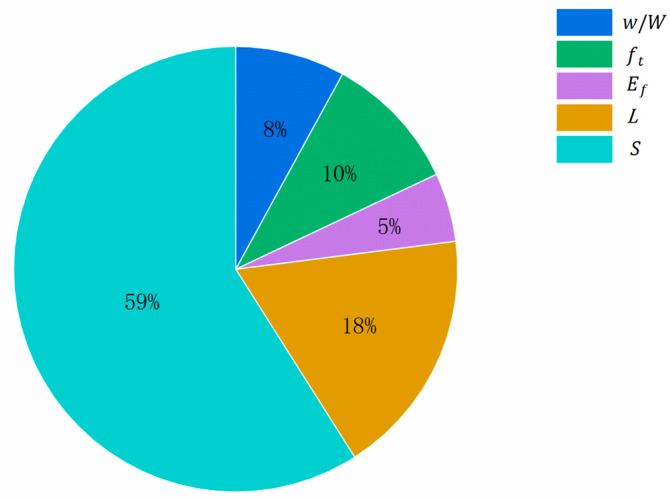
Variable importance of parameters in GEP-based prediction models.

To better understand the effect of different influences on fatigue life, the model was analyzed for the importance of variables. When calculating the importance of an input variable, the original input value was used first to predict the model, and the $R^{2}$ between the model output and the target calculated. Then, by randomly disrupting the input value of a certain variable and keeping the input value of other model variables unchanged, the reduction in $R^{2}$ between the model output and the target from the original $R^{2}$ of the model is calculated. Finally, the results of all the variables are normalized such that their sum is 1, thus allowing the importance of each variable to be obtained. The results are shown in Figure 8, where it can be seen that the order of parameter importance in the GEP model of this paper is $S(59\%)>L(18\%)>f_{t}(10\%)>w/W(8\%)>E_{f}(5\%)$, which is consistent with the results of the Pearson correlation analysis.

## 4. Comparative Analysis with Existing Models

The existing S–N curve shows the stress level *S* and fatigue life *N* expression, which has two main forms: single logarithm and double logarithm. Zhu et al. [8] considered the effect of concrete strength in addition to the stress level, Li [7] proposed a model for fatigue life prediction considering multiple factors, and Fathi et al. [12] modified Li’s model based on the collection of experimental data. To further evaluate the prediction effect of the FRP–concrete interface fatigue life model (GEP model) proposed in this paper, it is compared with several more common prediction models in the literature, the calculation formulas for which are shown in Table 5. The evaluation indexes of GEP and other models are calculated separately and listed in Table 6; i.e., for the model obtained using the EB reinforcement method, the data of all the EB reinforcement samples (108 sets) in the database of this paper are used to calculate the evaluation indexes, and for the model obtained using the NSM reinforcement method, the data of all the NSM reinforcement samples (111 sets) in the database of this paper are used to calculate the evaluation indexes.

As can be seen from Table 6, the coefficients of determination of the GEP model proposed in this paper are 0.841 and 0.762 for the EB FRP systems and NSM FRP systems, respectively, which are higher than the coefficients of determination of the other models and the statistical indices such as root-mean-squared error, which are lower than those of the other models. Among them, the stress level using the fatigue load magnitude and the stress magnitude of these two forms of prediction model of the statistical indices are poor because the test load difference is large, resulting in the poor applicability of these two formulas. The use of relative load form is equivalent to the load normalization, which helps to improve the model’s applicability, but the application of this form of relative loading is very critical to choose a suitable prediction formula for the bond strength under static loading conditions. This indicates that the selection of parameters and the number of data in the modeling process have a significant impact on the model, and the GEP model can be optimized and improved by obtaining experimental data through tests to improve the prediction accuracy and the applicability of the model. 

**Figure 9:**
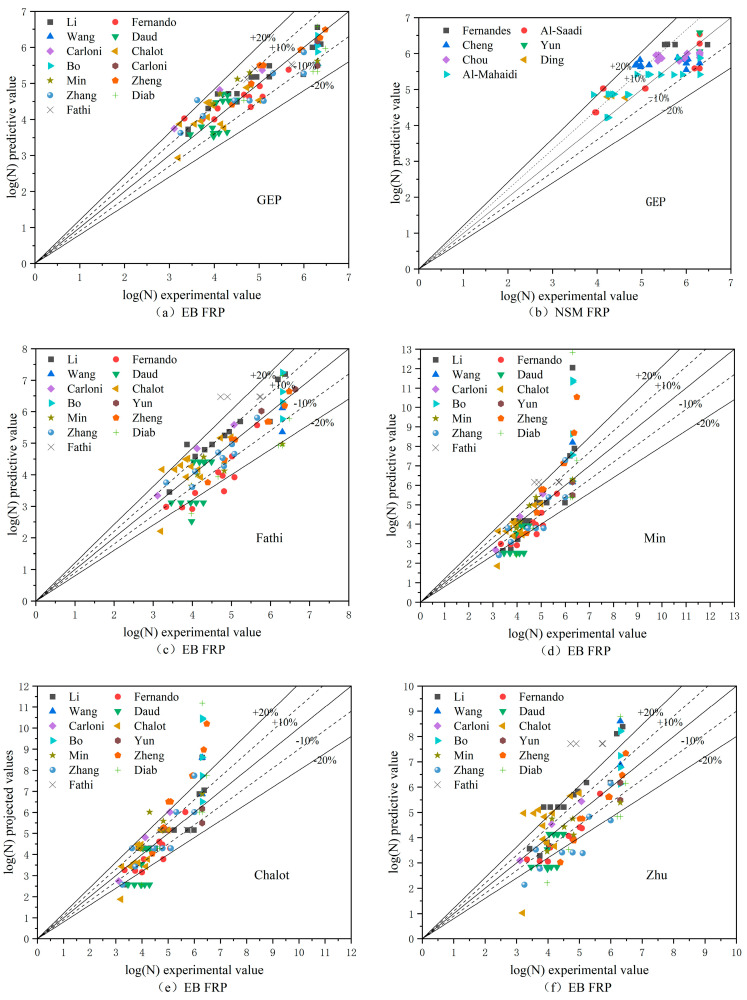

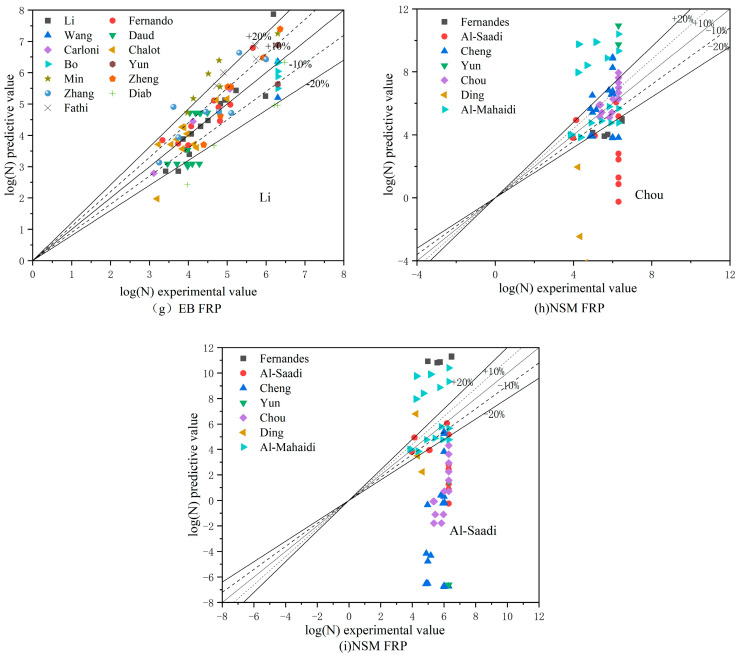
Diagram comparing the models for fatigue life prediction.

Figure 9 further gives the comparison results of the predicted and test values of each model. This figure intuitively shows that, based on the GEP model, most of the errors in the predicted interface fatigue life of the two reinforcement modes, EB FRP and NSM FRP, are within 10%. A small number of predictions exceeding 10% fall into the ±20% error limit, and only a small number of errors exceeding ±20% are in its vicinity, and those of the NSM FRP are slightly dispersed. The reason is that the test variables in the literature [13,28] involve the degree of roughness of the FRP, which leads to a large difference in the fatigue life of specimens with the roughness degree of FRP as the test variable. However, the overall fit between the predicted values and the test values is high. The model’s error is much smaller than that of the other models, which indicates that the model based on the programming of the gene expression shows high accuracy and good applicability in the prediction of fatigue life and has good application prospects.

## 5. Conclusions

The existing model of FRP–concrete fatigue life prediction has unstable prediction accuracy and limited generalization ability in its applications. Therefore, in this paper, we established a prediction model of FRP–concrete interface fatigue life that is applicable to two reinforcement methods, EB FRP and NSM FRP, based on the GEP. This paper presents the following conclusions.

1. Based on the results of the Pearson analysis of the database in this paper and the existing research results, five different input forms were selected to study their effects on the accuracy of fatigue life prediction. The optimal input form of the model was obtained, and the explicit expression of the fatigue life prediction model considering multiple factors was obtained.
2. The reasonableness of the model proposed in this paper is proven using variable sensitivity analysis and importance analysis. Among them, the fatigue life increases with the increase in concrete tensile strength and bond length and decreases with the increase in stress level. Further study is needed on the effects of the FRP-to-concrete width ratio (EB) and groove depth-to-width ratio (NSM) on fatigue life.
3. When comparing and analyzing the GEP model with the existing model, we found that the $R^{2}$ of the GEP model is higher than that of the existing model, and the statistical indices such as RMSE are lower than that of other models, while the prediction error is smaller. This shows that the GEP model proposed in this paper has a better prediction effect and provides a new idea for studying the fatigue life of the FRP–concrete interface.
4. The prediction model has a certain generalization ability, and the data can be expanded to improve the generalization and accuracy of the model.

## Figures and Tables

**Table 1:** Statistical parameters of the experimental data.

Parameter	Min	Max	Average	Median	Standard Deviation
$f_{t}\;(MPa)$	1.99	4.08	3.28	3.14	0.45
L (mm)	20	530	202.32	180	98.24
$A_{f\;}({mm}^{2})$	4.18	140	33.98	28	28.32
$E_{f\;}(GPa)$	60	264.86	186.18	212	44.23
w/W	0.17	6	1.91	1	2
$S_{max}$	0.3	0.9	0.64	0.66	0.12
$S_{min}$	0.04	0.33	0.12	0.1	0.06
$E_{f}A_{f}\;(kN)$	169.62	24,500	5032.21	3696	4533.35
∆S	0.12	0.80	0.52	0.5	0.13
$S_{a}$	0.19	0.51	0.38	0.39	0.07
*S*	0.04	0.4	0.2	0.21	0.08
log (N)	3.11	6.47	5.19	5.14	0.99

**Table 2:** Parameter settings of the fatigue life prediction model.

Parameter Types	Setting	Parameter Types	Setting
Population size	50	Gene Number	3
Head length	8	Chromosome length	45
Connection function	+	Mutation rate	0.00138
Gene transposition rate	0.00277	Gene recombination rate	0.00277
IS transposition rate	0.00546	RIS transposition rate	0.00546
One-point recombination rate	0.00277	Two-point recombination rate	0.00277

**Table 3:** Input form of the model for interface fatigue life prediction.

Model	Fatigue Life *log*(*N*)
A	$log(N)=f_{1}(f_{t},\;L,E_{f},A_{f},w/W,S)$
B	$log(N)=f_{2}(f_{t},L,E_{f}A_{f},w/W,S)$
C	$log(N)=f_{3}(f_{t},L,E_{f},w/W,S_{a})$
D	$log(N)=f_{4}(f_{t},L,E_{f},w/W,∆S)$
E	$log(N)=f_{5}(f_{t},L,E_{f},w/W,S)$

**Table 4:** Statistical indices for models with different input parameters.

Model	*R* ^2^	RMSE	MAE	RRSE	MAPE
Model A	0.751	0.504	0.411	0.499	0.083
Model B	0.734	0.528	0.431	0.523	0.089
Model C	0.648	0.604	0.499	0.598	0.103
Model D	0.623	0.621	0.496	0.614	0.103
Model E	0.819	0.423	0.352	0.426	0.071

**Table 5:** Model for FRP–concrete interface fatigue life prediction.

Reinforcement Method	Model and Year	Fatigue Life
EB	Fathi [12] (2023)	$\frac{∆S}{1-S_{a}}=1.916-0.0908\left\{\frac{\ln\left(N\right)}{\left(0.0021f_{c}+0.872)(1.094-0.382w/W\right)}\right\}$
	Min [11] (2020)	$\log\left(N\right)=-6.92-11.63\log\left(S/2\right)$
	Chalot [10] (2019)	$S_{max}=-0.058\log\left(N\right)+0.949$
	Zhu [8] (2016)	$\ln\left(N\right)=-31.646(∆S-0.8683)(0.0021f_{c}+0.8724)$
	Li [7] (2015)	$S_{max}=1-0.056\sqrt{2\left(1-r\right)}log(N){\left[\left(2.1\times 10^{-3}f_{c}+0.872)\times (1.094-0.382w/W)\times (1.3\times 10^{-3}L+0.779\right)\right]}^{-1}$
NSM	Chou [14] (2022)	$S_{n}=381.35033-29.41709log(N)$
	Al-Saadi [13] (2016)	$L_{R}=26.465-1.7587log(N)$

Notes: $r=S_{min}/S_{max}$, and $L_{R}=P_{max}-P_{min},S_{n}=\frac{L_{R}}{2A_{f}}$, the remaining parameters are explained in Table 1.

**Table 6:** Statistical indices for each model.

Reinforcement Method	Model	*R* ^2^	RMSE	MAE	RRSE	MAPE
EB	GEP	0.841	0.406	0.329	0.398	0.071
	Fathi [12]	0.697	0.681	0.545	0.654	0.115
	Min [11]	0.692	1.606	1.02	1.543	0.199
	Chalot [10]	0.758	1.355	0.916	1.302	0.178
	Zhu [8]	0.557	1.169	0.919	1.124	0.196
	Li [7]	0.627	1.208	0.799	1.161	0.177
NSM	GEP	0.762	0.439	0.373	0.504	0.070
	Chou [14]	0.151	7.396	5.031	8.461	1.017
	Al-Saadi [13]	0.007	5.964	5.019	6.823	0.926

## Data Availability

Data are contained within the article.

## Supplementary Materials

The following supporting information can be downloaded at: https://www.mdpi.com/article/10.3390/ma17030690/s1, Table S1: Experimental database.
