# Supplementary material for: Fatigue Life Prediction Model of FRP–Concrete Interface Based on Gene Expression Programming
Source: Materials (Basel). 2024 Jan 31;17(3):690. doi: 10.3390/ma17030690 (PMC10856217; doi:10.3390/ma17030690)
Supplement: Supplementary file 1 [file materials-17-00690-s001.zip › materials-2794657-supplementary.pdf]

Supplementary materials

Table S1

Reinforcement method	Author	Specimen number	f_t(MPa)	L(mm)	E_f(GPa)	w/W	S	$log(N)$
NSM	Jia-xuan, C	B2F32	3.040978	28	140.2	2	0.049152	6.30103
		B2F34	3.040978	28	140.2	2	0.055488	6.30103
		B2F36	3.040978	28	140.2	2	0.062208	6.30103
		B2F38	3.040978	28	140.2	2	0.069312	6.30103
		B2F40	3.040978	28	140.2	2	0.0768	6.30103
		B2F42.5_1	3.040978	28	140.2	2	0.0867	6.018435
		B2F42.5_2	3.040978	28	140.2	2	0.0867	6.30103
		B2F45_1	3.040978	28	140.2	2	0.0972	5.377239
		B2F45_2	3.040978	28	140.2	2	0.0972	5.322095
		B2F48_1	3.040978	28	140.2	2	0.110592	5.779539
		B2F48_2	3.040978	28	140.2	2	0.110592	5.779539
		B2F50_1	3.040978	28	140.2	2	0.12	5.662145
		B2F50_2	3.040978	28	140.2	2	0.12	5.662145
		B3L20	3.040978	20	140.2	2	0.0768	6.30103
		B3L24	3.040978	24	140.2	2	0.0768	6.30103
		B3L28	3.040978	28	140.2	2	0.0768	6.30103
	Fernandes	DPT1_F50	3.289387	60	169.5	3	0.10875	6.477121
		DPT2_F50	3.289387	60	169.5	3	0.10875	6.477121
		DPT3_F50	3.289387	60	169.5	3	0.10875	6.477121
		DPT1_F60	3.289387	60	169.5	3	0.13695	5.715874
		DPT2_F60	3.289387	60	169.5	3	0.14025	5.748963
		DPT3_F60	3.289387	60	169.5	3	0.1344	5.575188
	Al-Saadia	FS10E-60-1	3.594775	180	212	3	0.175	6.185502
		FS10E-60-2	3.594775	180	212	3	0.175	6.185388
		FS20E-60-1	3.594775	180	212	6	0.175	6.30103
		FS20E-60-2	3.594775	180	212	6	0.175	6.30103
		FR10E-60-1	3.594775	180	212	3	0.175	6.30103
		FR10E-60-2	3.594775	180	212	3	0.175	6.30103
		FR20E-60-1	3.594775	180	212	6	0.175	6.30103
		FR20E-60-2	3.594775	180	212	6	0.175	6.30103
		FS10E-67.5-1	3.594775	180	212	3	0.222813	4.144387
		FS10E-67.5-2	3.594775	180	212	3	0.222813	4.130784
		FS20E-67.5-1	3.594775	180	212	6	0.222813	6.30103
		FS20E-67.5-2	3.594775	180	212	6	0.222813	6.30103
		FR10E-67.5-1	3.594775	180	212	3	0.222813	5.090089
		FR10E-67.5-2	3.594775	180	212	3	0.222813	5.075799
		FR20E-67.5-1	3.594775	180	212	6	0.222813	6.30103
		FR20E-67.5-2	3.594775	180	212	6	0.222813	6.30103
		FS10E-75-1	3.594775	180	212	3	0.27625	3.98064
		FS10E-75-2	3.594775	180	212	3	0.27625	3.954677
		FS20E-75-1	3.594775	180	212	6	0.27625	6.30103
		FS20E-75-2	3.594775	180	212	6	0.27625	6.30103
		FR10E-75-1	3.594775	180	212	3	0.27625	4.132067
		FR10E-75-2	3.594775	180	212	3	0.27625	4.099819
		FR20E-75-1	3.594775	180	212	6	0.27625	6.30103
		FR20E-75-2	3.594775	180	212	6	0.27625	6.30103
		FS10E-82.5-1	3.594775	180	212	3	0.335313	3.324077
		FS10E-82.5-2	3.594775	180	212	3	0.335313	3.304059
		FS20E-82.5-1	3.594775	180	212	6	0.335313	5.417119
		FS20E-82.5-2	3.594775	180	212	6	0.335313	5.414094
		FR10E-82.5-1	3.594775	180	212	3	0.335313	3.907358
		FR10E-82.5-2	3.594775	180	212	3	0.335313	3.898944
		FR20E-82.5-1	3.594775	180	212	6	0.335313	5.953921
		FR20E-82.5-2	3.594775	180	212	6	0.335313	5.95271
		FR20E-90-1	3.594775	180	212	6	0.4	4.657629
		FR20E-90-2	3.594775	180	212	6	0.4	4.657027
	Cheng	R-RO-A1-1060-F-N-1	3.818353	250	117	1	0.172394	4.978655
		R-RO-A1-1060-F-N-2	3.818353	250	117	1	0.172212	4.898456
		R-RO-A1-1060-F-N-3	3.818353	250	117	1	0.172415	4.855967

		R-SC-A1-1060-F-N-1	3.818353	250	113	1	0.170098	5.166149
		R-SC-A1-1060-F-N-2	3.818353	250	113	1	0.172818	4.975818
		R-SC-A1-1060-F-N-3	3.818353	250	113	1	0.169786	4.880739
		R-SCSW-A1-1060-F-N-1b	3.818353	250	129	1	0.173339	6.30103
		R-SCSW-A1-1060-F-N-2	3.818353	250	129	1	0.172831	6
		R-SCSW-A1-1060-F-N-3	3.818353	250	129	1	0.173315	6
		S-RO-A1-1060-F-N-1	3.818353	250	168	1.666667	0.174438	4.963396
		S-RO-A1-1060-F-N-2	3.818353	250	168	1.666667	0.174167	5.929593
		S-RO-A1-1060-F-N-3	3.818353	250	168	1.666667	0.174715	6.036561
		S-RO-A2-1060-F-N-2	3.818353	250	168	1.666667	0.171776	5.806441
		S-RO-A2-1060-F-N-3	3.818353	250	168	1.666667	0.172143	6
		S-RO-A3-1060-F-N-1	3.818353	250	168	1.666667	0.174325	6
		S-RO-A3-1060-F-N-2	3.818353	250	168	1.666667	0.167361	6
		S-RO-A3-1060-F-N-3	3.818353	250	168	1.666667	0.212061	6
		S-RO-A4-1060-F-N-1	3.818353	250	168	1.666667	0.175428	6
		S-RO-A4-1060-F-N-2	3.818353	250	168	1.666667	0.176017	6
		S-RO-A4-1060-F-N-3	3.818353	250	168	1.666667	0.176309	6
	Yun	F-NSM-A	3.714518	100	257	1	0.09855	6.30103
		F-NSM-B	3.714518	100	257	1	0.19575	6.30103
		FC30-BL100	2.718011	100	165	2	0.262762	4.229707
	Ding	FC30-BL150	2.718011	150	165	2	0.270289	4.337898
		FC30-BL200	2.718011	200	165	2	0.271969	4.631941
		FS10C-60-1	3.040978	180	212	3	0.175	6.30103
		FS10C-60-2	3.040978	180	212	3	0.175	6.30103
		FS20C-60-1	3.040978	180	212	6	0.175	6.30103
		FS20C-60-2	3.040978	180	212	6	0.175	6.30103
		FR10C-60-1	3.040978	180	212	3	0.175	5.819661
		FR10C-60-2	3.040978	180	212	3	0.175	5.818086
		FR20C-60-1	3.040978	180	212	6	0.175	6.30103
		FR20C-60-2	3.040978	180	212	6	0.175	6.30103
		FS10C-67.5-1	3.040978	180	212	3	0.222813	5.716799
		FS10C-67.5-2	3.040978	180	212	3	0.222813	5.715443
		FS20C-67.5-1	3.040978	180	212	6	0.222813	5.182349
		FS20C-67.5-2	3.040978	180	212	6	0.222813	5.135129
		FR10C-67.5-1	3.040978	180	212	3	0.222813	5.423621
		FR10C-67.5-2	3.040978	180	212	3	0.222813	5.42281
	Al-Mahaidi	FR20C-67.5-1	3.040978	180	212	6	0.222813	5.908961
		FR20C-67.5-2	3.040978	180	212	6	0.222813	4.890443
		FR20IC-67.5-1	3.040978	180	212	6	0.222813	6.30103
		FR20IC-67.5-2	3.040978	180	212	6	0.222813	6.30103
		FS10C-75-1	3.040978	180	212	3	0.27625	4.708752
		FS10C-75-2	3.040978	180	212	3	0.27625	4.677917
		FS20C-75-1	3.040978	180	212	6	0.27625	4.287667
		FS20C-75-2	3.040978	180	212	6	0.27625	4.242144
		FR10C-75-1	3.040978	180	212	3	0.27625	3.913761
		FR10C-75-2	3.040978	180	212	3	0.27625	3.827951
		FR20C-75-1	3.040978	180	212	6	0.27625	4.383079
		FR20C-75-2	3.040978	180	212	6	0.27625	4.343783
		FS10C-82.5-1	3.040978	180	212	3	0.335313	4.253919
		FS10C-82.5-2	3.040978	180	212	3	0.335313	4.215611
		FR20C-82.5-1	3.040978	180	212	6	0.335313	4.102399
		FR20C-82.5-2	3.040978	180	212	6	0.335313	4.080049
		F-0.45-1	3.764337	150	210	0.5	0.100238	6.30103
		F-0.45-2	3.764337	150	210	0.5	0.100238	6.30103
		F-0.45-3	3.764337	150	210	0.5	0.100238	6.30103
	Fuling, W	F-0.55-1	3.764337	150	210	0.5	0.149738	6.30103
		F-0.55-2	3.764337	150	210	0.5	0.149738	6.30103
		F-0.55-3	3.764337	150	210	0.5	0.149738	6.30103
EB		DS-F1	2.781967	152	230	0.2	0.29925	3.11059
	Carlon	DS-F2	2.781967	152	230	0.2	0.2132	4.120311
		DS-F3	2.781967	152	230	0.2	0.16875	5.068167
		F-100-2	2.923261	100	165	0.333333	0.091908	6.30103
		F-150-2	2.923261	150	165	0.333333	0.148571	6.30103
	Bo, W	F-200-2	2.923261	200	165	0.333333	0.053181	6.30103
		F-150-1	2.923261	150	165	0.333333	0.11375	6.30103

	F-150-3	2.923261	150	165	0.333333	0.054102	6.30103
Zhang	C13HS-F70	1.991578	400	156	0.333333	0.24	5.105323
	C21HS-F60	2.222969	400	156	0.333333	0.175	6
	C21HS-F70	2.222969	400	156	0.333333	0.24	4.45011
	C21HS-F70-T	2.222969	400	156	0.333333	0.24	4.787553
	C21HS-F75-T	2.222969	400	156	0.333333	0.27625	3.746089
	C21HS-F80	2.222969	400	156	0.333333	0.315	3.250176
	C36HS-F50	3.015584	400	156	0.333333	0.12	6
	C36HS-F60	3.015584	400	156	0.333333	0.175	5.312124
	C36HS-F70	3.015584	400	156	0.333333	0.24	3.617525
Yun	F-EB-A	3.215878	100	257	0.25	0.09625	6.30103
	F-EB-B	3.235562	100	257	0.25	0.15355	6.30103
Daud	F2	2.781967	300	114.9	0.25	0.23375	4.037426
	F3	2.781967	300	264.86	0.25	0.23375	4.177536
	F4	2.781967	300	128.46	0.25	0.23375	4.318877
	F5	2.781967	300	138.35	0.25	0.23375	4.491362
	F6	2.781967	300	220.6	0.25	0.23375	4.291147
	F7	2.781967	300	128.46	0.25	0.23375	4.187521
	F9	2.781967	300	114.9	0.25	0.30875	3.462398
	F10	2.781967	300	264.86	0.25	0.30875	3.70757
	F11	2.781967	300	128.46	0.25	0.30875	4.100371
	F12	2.781967	300	138.35	0.25	0.30875	4.183301
	F13	2.781967	300	220.6	0.25	0.30875	3.956649
	F14	2.163287	300	128.46	0.25	0.30875	3.982271
Zheng	FA-1	3.500456	250	226	0.5	0.252551	4.395868
	FA-2	3.500456	250	226	0.5	0.252551	4.395885
	FA-3	3.500456	250	226	0.5	0.252551	4.395903
	FB-1	3.500456	250	226	0.5	0.204566	4.825588
	FB-2	3.500456	250	226	0.5	0.204566	4.825588
	FC-1	3.500456	250	226	0.5	0.161633	5.015967
	FC-2	3.500456	250	226	0.5	0.161633	5.095455
	FD-1	3.500456	250	226	0.5	0.12375	5.926881
	FD-2	3.500456	250	226	0.5	0.12375	5.959055
	FE-1	3.500456	250	226	0.5	0.090918	6.362844
	FE-2	3.500456	250	226	0.5	0.090918	6.362844
	FF-1	3.500456	250	226	0.5	0.063138	6.477121
Fernandob	SC60-1	3.984809	300	175	0.25	0.16875	5.658774
	SC60-2	3.984809	300	178.5	0.25	0.23375	5.079362
	MC60-1	3.984809	300	160.2	0.25	0.2552	4.814248
	MC60-2	3.984809	300	158.6	0.25	0.2816	3.334454
	MC60-3	3.984809	300	160.5	0.25	0.226512	4.662758
	SC50-3	3.528743	300	176.5	0.25	0.285566	3.999565
	MC50-1	3.528743	300	157.7	0.25	0.258	4.073718
	MC50-2	3.528743	300	157	0.25	0.2052	5.015779
	MC50-3	3.528743	300	160.5	0.25	0.231438	4.778151
	MC50-4	3.528743	300	160.5	0.25	0.28288	3.740363
Diad	F-80-1	3.040978	200	240	0.5	0.315	3.973636
	F-80-2	3.040978	200	240	0.5	0.315	3.973636
	F-80-3	3.040978	200	240	0.5	0.315	3.973636
	F-70-1	3.040978	200	240	0.5	0.24	4.659155
	F-70-2	3.040978	200	240	0.5	0.24	4.659155
	F-70-3	3.040978	200	240	0.5	0.24	4.659155
	F-60-1	3.040978	200	240	0.5	0.175	6.20412
	F-60-3	3.040978	200	240	0.5	0.175	6.30103
	F-50-1	3.040978	200	240	0.5	0.12	6.477121
	F-50-2	3.040978	200	240	0.5	0.12	6.477121
	F-30-1	3.040978	200	240	0.5	0.04	6.30103
	F-30-2	3.040978	200	240	0.5	0.04	6.301031
Min	FT-0	2.565751	530	175	0.666667	0.14625	6.30103
	FT-2	2.565751	530	175	0.666667	0.190313	4.815026
	FT-3	2.565751	530	175	0.666667	0.20625	4.816977
	FT-4	2.565751	530	175	0.666667	0.24	3.97511
	FT-6	2.565751	530	175	0.666667	0.225	4.127655
Chalot	A-F2-1	3.040978	200	60	0.357143	0.25665	4.216377

	A-F3-1	3.040978	200	60	0.357143	0.18705	4.997784
	A-F3-2	3.040978	200	60	0.357143	0.24745	3.557387
	A-F3-3	3.040978	200	60	0.357143	0.24745	4.146686
	A-F3-4	3.040978	200	60	0.357143	0.24745	3.230193
	A-F3-5	3.040978	200	60	0.357143	0.24	3.705436
	B–F1-4	2.938748	200	105	0.357143	0.35155	3.197005
	B–F2-4	3.141518	200	105	0.357143	0.25935	3.848374
	B–F3-1	2.990082	200	105	0.357143	0.232	3.970951
	B–F3-2	3.141518	200	105	0.357143	0.22525	3.841422
	B–F3-3	2.990082	200	105	0.357143	0.225	3.889918
	B–F3-4	3.141518	200	105	0.357143	0.1892	4.723267
Carloni	DDS-FT-1	3.141518	152	230	0.2	0.261	3.971601
	DDS-FT-2	3.141518	152	230	0.2	0.2976	3.741388
	DDS-FT-3	3.141518	152	230	0.2	0.26845	4.02387
Ke, L	A-1	4.08163	160	225	0.25	0.30195	3.414973
	A-2	4.08163	160	226	0.25	0.22295	4.50515
	A-3	4.08163	160	227	0.25	0.1848	5.22763
	A-4	4.08163	160	228	0.25	0.1148	6.190332
	A-5	4.08163	160	229	0.25	0.1066	6.376577
	A-6	4.08163	160	230	0.25	0.0468	6.30103
	B-1	2.797842	160	231	0.25	0.1848	4.945961
	B-2	2.228893	160	232	0.25	0.1848	4.826075
	C-1	4.08163	160	233	0.36	0.22295	4.315551
	C-2	4.08163	160	234	0.5	0.22295	4.072985
	D-1	4.08163	60	235	0.25	0.22295	3.863323
	D-2	4.08163	240	236	0.25	0.22295	5.854243
	E-1	4.08163	240	236	0.25	0.1848	5.986772
Fathi	CL-50-25	3.760236	50	165	0.17	0.15	4.724112
	CL-100-25	3.760236	100	165	0.17	0.15	4.901371
	CL-150-25	3.760236	150	165	0.17	0.15	5.747777
	CL-200-25	3.760236	200	165	0.17	0.15	5.722946
